# Effects of Oral Resveratrol Supplementation on Inflammatory and Cardiometabolic Outcomes in Adults: Protocol for a Systematic Review and Meta-Analysis

**DOI:** 10.2196/101301

**Published:** 2026-10-27

**Authors:** Andrea Herrera-Santelices, Josefa Camila Núñez Rivas, Francisca P Rubio-Molina, Ileana González Bonet

**Affiliations:** 1 Departamento de Ciencias Preclínicas, Facultad de Medicina, Universidad Católica del Maule, Avda San Miguel 3605, Talca, Maule, Chile, 56 712968000; 2 Servicio de Medicina Física y Rehabilitación, Hospital San Juan de Dios de Curicó, Servicio de Salud del Maule, Curicó, Maule, Chile; 3 Carrera de Medicina, Facultad de Medicina, Universidad Católica del Maule, Talca, Maule, Chile; 4 Sistema de Bibliotecas, Universidad Católica del Maule, Talca, Maule, Chile

**Keywords:** resveratrol, inflammatory biomarkers, cardiometabolic outcomes, systematic review, meta-analysis, randomized controlled trial

## Abstract

**Background:**

Resveratrol is a naturally occurring polyphenol found primarily in grapes, berries, peanuts, and red wine. Experimental evidence suggests that resveratrol modulates multiple biological pathways involved in oxidative stress, inflammation, insulin signaling, endothelial function, and metabolic homeostasis. However, findings from randomized controlled trials remain inconsistent, and previous evidence syntheses have reported substantial clinical and methodological heterogeneity.

**Objective:**

This is a protocol for a systematic review and meta-analysis that will provide an updated synthesis of randomized controlled trials evaluating the effects of oral resveratrol supplementation on inflammatory biomarkers and cardiometabolic outcomes in adults while exploring potential methodological and biological sources of heterogeneity.

**Methods:**

This protocol was developed according to the PRISMA-P (Preferred Reporting Items for Systematic Reviews and Meta-Analyses Protocols) statement and prospectively registered in PROSPERO. Randomized controlled trials comparing oral resveratrol supplementation with placebo, other nutritional supplements, or no intervention in adults will be eligible. Literature searches will be conducted in the PubMed, Cochrane CENTRAL, Scopus, Web of Science, and SciELO databases. Two reviewers will independently perform study selection, data extraction, and risk-of-bias assessment using the Cochrane risk of bias 2 tool. The certainty of evidence will be assessed using the GRADE (Grading of Recommendations Assessment, Development and Evaluation) approach.

**Results:**

During peer review, the search strategy was refined before initiation of the formal review. As of August 2026, the formal systematic review had not yet commenced. The literature search and study selection are scheduled to begin on September 1, 2026, and the systematic review is expected to be completed by April 2027, with dissemination of the findings anticipated thereafter.

**Conclusions:**

This systematic review and meta-analysis protocol is expected to provide an updated and transparent synthesis of the available randomized evidence regarding the effects of oral resveratrol supplementation on inflammatory biomarkers and cardiometabolic outcomes in adults.

## Introduction

Resveratrol (trans-3,5,4′-trihydroxystilbene) is a naturally occurring polyphenol belonging to the stilbene family, characterized by a C6–C2–C6 chemical structure with 3 hydroxyl groups that confer relevant antioxidant properties [1,2]. In the plant kingdom, resveratrol is synthesized in response to various environmental stressors, including fungal infection, UV radiation, and mechanical injury [2]. This compound is distributed across a wide range of plant species, with particularly high concentrations in the skin of red grapes and in other dietary sources such as berries, peanuts, and red wine [1,2].

Biomedical interest in resveratrol increased following the publication of the French paradox hypothesis. In 1992, Renaud and de Lorgeril [3] associated regular and moderate consumption of red wine with a lower incidence of coronary heart disease, suggesting that wine polyphenols could contribute to this protective effect. Since then, resveratrol has become one of the most extensively investigated dietary polyphenols. Experimental and clinical research has demonstrated its anti-inflammatory and antioxidant properties, as well as its potential to improve vascular function and other mechanisms involved in cardiometabolic health [1,4-6].

Previous meta-analytic evidence from 21 clinical trials involving participants with overweight and obesity showed that resveratrol supplementation significantly reduced total cholesterol levels, systolic blood pressure, and fasting glucose, with more pronounced effects observed at daily doses exceeding 300 mg [7]. Similarly, subsequent meta-analyses reported reductions in body weight, BMI, waist circumference, and fat mass following resveratrol supplementation [8]. However, evidence regarding its anti-inflammatory effects remains inconsistent, with some studies reporting reductions in C-reactive protein and interleukin-6 (IL-6), whereas others observed no significant changes in inflammatory biomarkers [9].

Several systematic reviews and meta-analyses have evaluated the effects of resveratrol supplementation on specific inflammatory, anthropometric, glycemic, or lipid-related outcomes, frequently in selected clinical populations [10]. Nevertheless, substantial heterogeneity persists across randomized controlled trials regarding resveratrol dosage, intervention duration, formulation, baseline metabolic status, and outcome selection [11]. In addition, recent evidence concerning resveratrol bioavailability, metabolism, and interindividual variability has not been comprehensively integrated into previous evidence syntheses [4]. Consequently, the overall consistency and clinical relevance of oral resveratrol supplementation for inflammatory and cardiometabolic parameters in adults remain uncertain [4,12,13].

Therefore, this systematic review aims to provide an updated and comprehensive synthesis of randomized controlled trials evaluating the effects of oral resveratrol supplementation on inflammatory biomarkers and cardiometabolic outcomes in adults, while considering potential methodological and biological sources of heterogeneity that may contribute to inconsistent findings across studies [4,11]. Inflammatory biomarkers were prespecified as the primary outcomes because they directly reflect the biological pathways through which resveratrol is hypothesized to exert its effects [6], whereas cardiometabolic parameters were prespecified as secondary outcomes because they represent clinically relevant downstream outcomes that have been evaluated across multiple randomized trials [4,10].

The synthesis of available human evidence will strengthen understanding of the translational relevance of resveratrol and its potential role as an adjunct in the prevention or management of cardiometabolic diseases and its effect on inflammatory biomarkers. Ultimately, this work will contribute to evidence-based guidance for clinical practice and future research on polyphenol-based nutritional interventions [14].

## Methods

### Registration

This systematic review protocol was developed in accordance with the PRISMA-P (Preferred Reporting Items for Systematic Reviews and Meta-Analyses Protocols) guidelines to ensure rigorous and transparent methodological planning [15]. The PRISMA-P checklist is available in Checklist 1. The protocol was prospectively registered in PROSPERO (CRD420251178811) [16], clearly defining the objectives, eligibility criteria, and analytical methods. Any relevant amendments to the original protocol will be appropriately documented, dated, justified, and reported both in the final manuscript and the PROSPERO registry, thereby ensuring the traceability and transparency of the research process.

The research question addressed by this systematic review and meta-analysis is as follows: What are the effects of oral resveratrol supplementation, compared with placebo, other nutritional supplements, or no intervention, on inflammatory markers and cardiometabolic parameters in adults?

### Eligibility Criteria

The eligibility criteria for this systematic review were defined according to the population, intervention, comparator, and outcome (PICO) framework and summarized in Table 1, with further details provided in the subsequent sections [17].

**Table 1.** Population, intervention, comparator, and outcome (PICO) criteria.

Component	Description
Population	Adult human participants, with or without cardiometabolic diseases
Intervention	Oral resveratrol supplementation
Comparator	Placebo, other oral nutritional supplements, or no intervention
Outcomes	Primary outcomes: inflammatory biomarkers, including IL-6^a^, TNF-α^b^, and hs-CRP^c^ Secondary outcomes: other inflammatory and oxidative stress–related biomarkers, including IL-9^d^, total antioxidant capacity, ascorbic acid, and advanced glycation end products; cardiometabolic parameters, including blood pressure, fasting glucose, fasting insulin, HOMA-IR^e^, hemoglobin A_1c_, lipid profile, and hepatic profile; anthropometric and body composition outcomes, including BMI, body weight, waist circumference, fat mass, and muscle mass; oxidative damage markers, including malondialdehyde and DNA damage (comet assay)
Study design	Randomized controlled trials with a parallel-group design conducted in human participants

a IL-6: interleukin-6.

b TNF-α: tumor necrosis factor alpha.

c hs-CRP: high-sensitivity C-reactive protein.

d IL-9: interleukin-9.

e HOMA-IR: Homeostasis Model Assessment of Insulin Resistance.

### Types of Population

Studies reporting outcomes of interest in adult human populations, with or without cardiometabolic diseases, such as obesity, metabolic syndrome, type 2 diabetes mellitus, hypertension, dyslipidemia, or cardiovascular disease, will be included. Studies will be eligible regardless of sex, ethnicity, or geographical region. Studies involving pregnant or lactating women, as well as children and adolescents, will be excluded from this review.

### Type of Intervention

In this systematic review, the intervention of interest is oral resveratrol supplementation, administered orally as capsules, tablets, or other equivalent formulations, either as an isolated compound or as a standardized nutritional supplement. No strict restrictions will be applied regarding the daily dose or duration of the intervention, if these parameters are clearly reported, to capture the heterogeneity of available randomized controlled trials and allow a comprehensive assessment of their effects.

### Types of Comparators

Eligible studies must evaluate resveratrol supplementation as the primary intervention, compared with placebo, other oral nutritional supplements, or no intervention, in adult human populations.

### Types of Outcomes

Outcomes are categorized as primary and secondary.

The primary outcomes will comprise 3 inflammatory biomarkers: IL-6, tumor necrosis factor α, and high-sensitivity C-reactive protein.

Secondary outcomes will include other inflammatory and oxidative stress-related biomarkers: interleukin-9, total antioxidant capacity, ascorbic acid, and advanced glycation end products; cardiometabolic parameters, including blood pressure, fasting glucose and insulin, insulin resistance estimated by the homeostasis model assessment of insulin resistance, hemoglobin A_1c_, lipid profile, and hepatic profile. Additional secondary outcomes will include anthropometric and body composition measures, such as BMI, body weight, waist circumference, fat mass, and muscle mass, as well as markers of oxidative damage, including malondialdehyde and DNA damage assessed by the comet assay.

The predefined primary outcomes will be prioritized in the abstract, summary of findings tables, and overall interpretation of the review, whereas secondary outcomes will be presented and interpreted separately according to their respective outcome domains.

### Types of Studies

The inclusion and exclusion criteria applied to study eligibility are summarized in Textbox 1. Only parallel-group randomized controlled trials meeting the predefined eligibility criteria will be included.

Crossover randomized trials will be excluded because several predefined primary and secondary outcomes (eg, inflammatory biomarkers, hemoglobin A_1c_, anthropometric measures, and other cardiometabolic parameters) may remain affected by residual treatment effects despite a washout period, increasing the risk of carryover effects [18]. Furthermore, no universally accepted washout duration has been established for resveratrol across the broad range of outcomes evaluated in this review. Restricting eligibility to parallel-group randomized controlled trials therefore improves methodological consistency and the comparability of pooled estimates.

Textbox 1. **Inclusion and exclusion criteria for study eligibility.**

**Inclusion criteria**

- Randomized controlled trial with a parallel-group design
- Studies clearly reporting the intervention, comparator, and at least one predefined outcome of interest
- Studies published in English

**Exclusion criteria**

- Nonrandomized trials
- Observational studies (eg, cohort, case-control, and cross-sectional studies)
- Crossover randomized studies
- Case reports, narrative or systematic reviews, and meta-analyses
- Editorials, commentaries, letters to the editor, conference abstracts or proceedings, and protocols
- Modeling or simulation studies

### Search Strategy and Databases

A comprehensive and systematic search will be conducted from database inception in MEDLINE via PubMed, the Cochrane CENTRAL, Scopus, Web of Science Core Collection, and SciELO databases. ClinicalTrials.gov and the World Health Organization International Clinical Trials Registry Platform will also be searched to identify ongoing, completed, or unpublished trials. No restrictions regarding publication year will be applied, and only studies published in English will be eligible. The restriction to English-language publications was prespecified in the PROSPERO registration and was applied for pragmatic reasons to ensure consistent full-text eligibility assessment and data extraction within the language capabilities and resources available to the review team. SciELO was retained as a search source because it indexes English-language publications from Latin American and other settings that may not be adequately represented in the other databases searched.

Following peer review, the electronic search strategy was comprehensively redesigned to maximize sensitivity and minimize the risk of missing potentially eligible randomized controlled trials. The revised strategy combines terms related to resveratrol with randomized controlled trial identification methods appropriate for each information source. Outcome-related terms, including inflammatory biomarkers and cardiometabolic outcomes, will not be included in the electronic search. Eligibility regarding the predefined outcomes will instead be assessed during title and abstract screening and subsequently confirmed during full-text review.

The complete prespecified database-specific search strategies, including controlled vocabulary, free-text terms, Boolean operators, and randomized controlled trial identification methods appropriate for each information source, are presented in Multimedia Appendix 1. Any technical adaptations required by changes to a database interface will be documented, dated, and reported without modifying the underlying search concepts or eligibility criteria.

### Study Selection

All records retrieved from the electronic database searches will be imported into reference management software, and duplicate records will be removed prior to screening. Subsequently, 2 reviewers will independently screen the titles and abstracts of all identified studies to assess eligibility according to the predefined inclusion and exclusion criteria. The full texts of all studies that pass the initial screening stage will be independently assessed by 2 reviewers to confirm eligibility. A list of excluded studies with reasons for exclusion will be provided as supplementary material.

### Data Extraction

Data will be extracted independently from published articles and reports by at least 2 reviewers using a predefined Microsoft Excel data extraction form. The following data will be extracted: (1) general study characteristics: first author, year of publication, country, study design, setting, sample size (per group and total), number of study arms, and duration of follow-up; (2) population characteristics: mean age, sex distribution, baseline health status (eg, presence of obesity, type 2 diabetes, hypertension, dyslipidemia, or cardiovascular disease), inclusion and exclusion criteria, and baseline values of relevant outcomes; (3) intervention characteristics: formulation of resveratrol (eg, capsule or tablet), daily dose (mg/day), frequency of administration, duration of the intervention, and whether resveratrol was administered as a single compound or as part of a combined supplement; (4) comparator characteristics: type of comparator (placebo, other nutritional supplement, or no intervention) and relevant details of its composition and administration; (5) outcomes: for all predefined primary and secondary outcomes, the following data will be extracted: outcome definition and measurement method, units of measurement, time points assessed, baseline and postintervention values (means and SDs), change-from-baseline values when available, and number of participants analyzed per outcome; (6) methodological characteristics: information relevant to risk-of-bias assessment (eg, randomization procedures, allocation concealment, blinding, attrition, and outcome reporting); and (7) additional data: funding sources, declarations of conflicts of interest, and any information relevant to the interpretation of results.

A predefined process will be used to resolve any discrepancies: discrepancies between reviewers will be resolved through discussion, and if consensus cannot be reached, a third reviewer will be consulted. When required data are not available in the published reports, study authors will be contacted to obtain missing data.

### Risk-of-Bias Assessment

The risk of bias of the included studies will be assessed using the Cochrane risk of bias 2 (RoB 2) tool [19]. This tool is specifically designed for randomized controlled trials and evaluates bias across key domains, including bias arising from the randomization process, deviations from intended interventions, missing outcome data, measurement of the outcome, and selection of the reported result.

The risk-of-bias assessment will be conducted independently by at least 2 reviewers, using the predefined process to resolve any disagreements described previously. No additional information will be sought from study investigators in cases where required information is unclear or unavailable in the published reports. The results of the risk-of-bias assessment will be presented using both tabular summaries and graphical representations. Overall risk-of-bias judgments will be transparently reported and considered when interpreting the results of the review.

### Strategy for Data Synthesis

A quantitative synthesis (meta-analysis) will be conducted if at least 2 studies meet the inclusion criteria and provide data that are sufficiently comparable regarding participant characteristics, intervention, comparator, and outcome definitions, according to the prespecified clinical and methodological criteria described below. This approach aims to ensure that pooled estimates remain clinically meaningful and interpretable. If these criteria are not met, a structured narrative synthesis will be conducted and organized according to the predefined primary and secondary outcomes, following the principles of the Synthesis Without Meta-analysis (SWiM) reporting guideline [20,21].

For the purposes of this review, studies will be considered sufficiently comparable for quantitative synthesis when they evaluate clinically compatible adult populations, investigate orally administered resveratrol, include the same comparator category (placebo, active comparator, or no intervention or usual care), and report the same predefined outcome using compatible measurement methods [20]. Clinical differences in resveratrol dosage, intervention duration, formulation, and participants’ baseline health status will not automatically preclude quantitative synthesis; instead, these factors will be explored through the predefined subgroup and meta-regression analyses when sufficient data are available. When these prespecified comparability criteria are not met, results will be synthesized narratively.

When at least 2 studies are available within the same comparator category, meta-analyses will be conducted separately by comparator type (placebo, active comparator, or no intervention or usual care), because these comparisons address different clinical questions and analyzing these comparisons separately improves the clinical interpretability of pooled estimates [20].

The meta-analysis will be performed using RevMan (version 5.4; Cochrane) [22] and statistical software (eg, SPSS; IBM) when more advanced analyses are required. The effects of the intervention will be compared with those of the control group. For continuous outcomes, when studies report outcomes using the same measurement units, results will be expressed as weighted mean differences with 95% CIs. When the same outcome is measured using different but comparable measurement scales or units across studies, standardized mean differences with corresponding 95% CIs will be calculated [20]. Biologically distinct outcomes will not be combined in the same meta-analysis. When available, change-from-baseline values will be preferentially used; however, postintervention values will also be included and combined using appropriate methods in accordance with Cochrane recommendations [20].

When outcome data are missing or incompletely reported, the corresponding authors of the original studies will be contacted to request additional information. If the requested information cannot be obtained, studies will not be excluded solely because of incomplete outcome reporting. Instead, the available published data will be synthesized using the prespecified methods whenever possible, and the potential impact of incomplete reporting will be considered in the risk-of-bias assessment and sensitivity analyses. No statistical imputation of missing outcome data will be performed by the review authors [20].

Heterogeneity among studies will be assessed using the Q statistic and the *I*^2^ statistic, interpreted as low (*I*^2^≤25%), moderate (*I*^2^=25.1%‐50%), or high (*I*^2^>50%) [23]. Given the expected clinical and methodological heterogeneity, a random-effects model will be used as the primary analytical approach [20].

Where sufficient data are available, predefined subgroup and meta-regression analyses will be conducted according to resveratrol dosage, intervention duration, formulation, and baseline health status of participants (eg, healthy adults, obesity, metabolic syndrome, type 2 diabetes mellitus, hypertension, dyslipidemia, or cardiovascular disease) to explore potential methodological and biological sources of heterogeneity [20]. Meta-regression analyses will only be performed when at least 10 studies are available for the covariate of interest and will be conducted using 1 explanatory variable at a time [24]. Because meta-regression is based on study-level characteristics, any observed associations will be interpreted cautiously as exploratory findings and may be influenced by ecological confounding or residual between-study differences [24].

Sensitivity analyses will be performed to assess the robustness of the results by excluding studies at high risk of bias, studies with substantial missing outcome data, and potential outliers [20]. For trials with multiple intervention arms, groups will be combined or appropriately split according to Cochrane Handbook recommendations to avoid double counting [19]. Publication bias will be assessed using funnel plots and the Egger regression test when at least 10 studies are available [20,25].

### Certainty Assessment

The certainty of the evidence will be assessed using the GRADE (Grading of Recommendations Assessment, Development and Evaluation) approach [26] with GRADEpro GDT software by evaluating risk of bias, inconsistency, indirectness, imprecision, and publication bias. Two reviewers will independently assess the certainty of the evidence for each outcome. The certainty of the evidence will be rated as high, moderate, low, or very low for each primary and secondary outcome. Downgrading decisions will follow established GRADE guidance and will be justified and transparently reported [26]. Summary of findings tables will be generated for all primary outcomes, presenting the magnitude of effect, number of participants and studies, and the corresponding certainty ratings. Where appropriate, additional tables with summaries of findings will be provided for key secondary outcomes [26].

### Ethical Considerations

Ethics approval was not required because this study is a protocol for a systematic review and meta-analysis that will use data exclusively from previously published studies. No individual participant data will be collected, and no direct contact with human participants will occur. The review will follow predefined eligibility criteria, standardized methods for study selection, data extraction, risk-of-bias assessment, and evidence certainty evaluation.

## Results

During the peer-review process, the search strategy was refined to improve its sensitivity and comprehensiveness before initiation of the formal systematic review. At the time of manuscript revision in August 2026, formal literature searching, study selection, eligibility assessment, data extraction, and risk-of-bias assessment had not yet commenced. The planned study identification and selection process is presented prospectively in Figure 1. As the systematic review has not yet been conducted, numerical values in the PRISMA (Preferred Reporting Items for Systematic Reviews and Meta-Analyses) flow diagram are reported as “n=to be determined” and will be completed during the conduct of the systematic review. The review is expected to begin after finalization of the updated protocol on September 1, 2026, with completion anticipated by April 2027 and dissemination of the findings thereafter.

**Figure 1.**
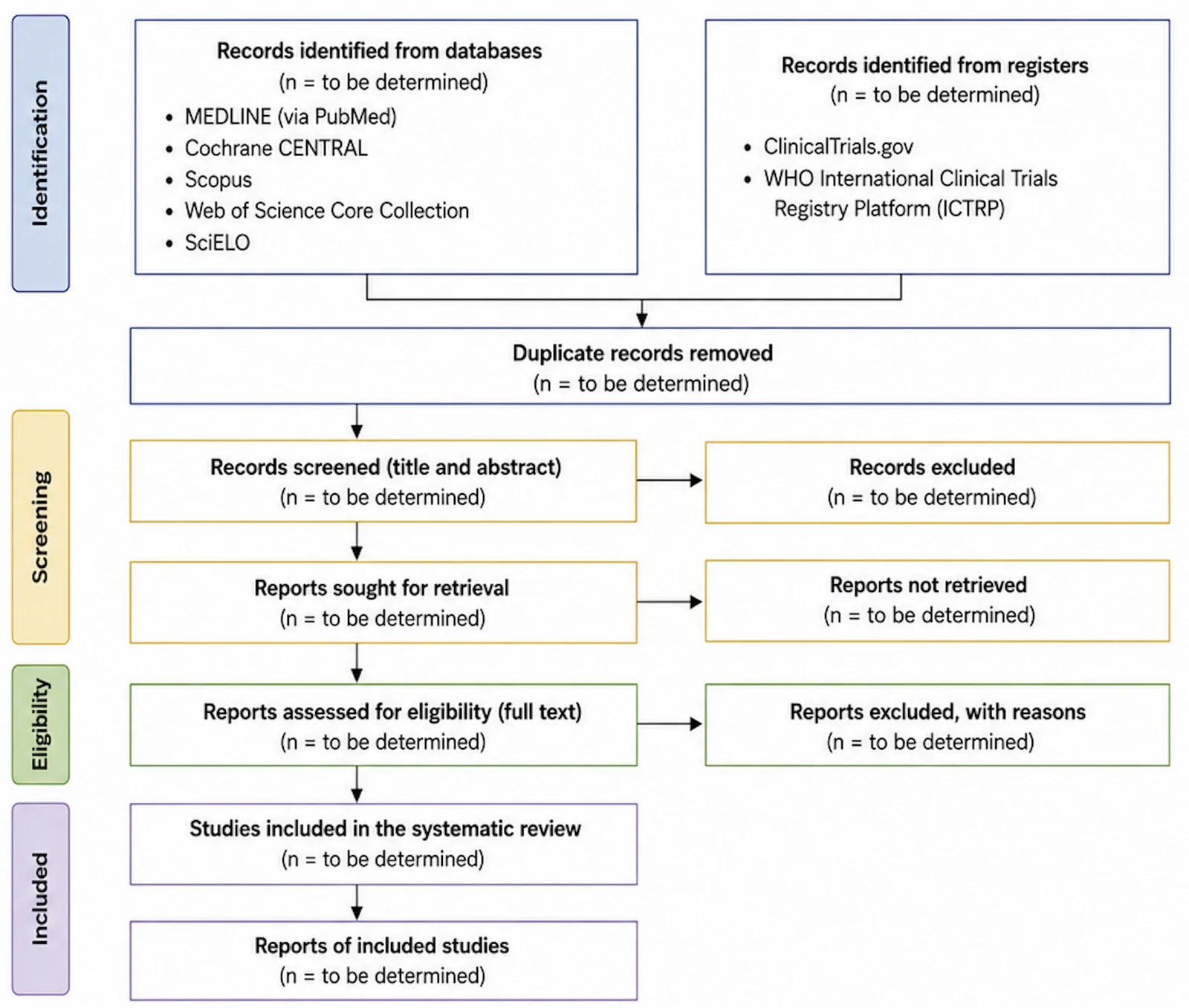
Prospective PRISMA (Preferred Reporting Items for Systematic Reviews and Meta-Analyses) flow diagram for the planned study identification and selection process. Numerical values (n) will be added during the systematic review. WHO: World Health Organization.

## Discussion

This systematic review and meta-analysis is expected to provide a comprehensive and up-to-date synthesis of randomized evidence evaluating the effects of oral resveratrol supplementation on inflammatory biomarkers and cardiometabolic outcomes in adults. Specifically, the review is expected to clarify the magnitude, consistency, and certainty of the available evidence while identifying potential sources of clinical and methodological heterogeneity that may explain divergent findings across studies. Given that previous randomized controlled trials have reported inconsistent findings and substantial differences in study populations, intervention characteristics, and reported outcomes [8,10,27], no assumptions are made regarding the direction or magnitude of the anticipated effects, which will be determined through the prespecified evidence synthesis and certainty assessment.

Previous systematic reviews and meta-analyses have evaluated specific inflammatory biomarkers, cardiometabolic outcomes, or selected patient populations; however, important heterogeneity persists regarding study populations, intervention characteristics, outcome definitions, and certainty of evidence [4,8,10,28]. Therefore, an updated systematic review of randomized controlled trials using predefined eligibility criteria and contemporary methodological standards remains warranted. By integrating evidence across these outcomes and systematically exploring potential methodological and biological sources of heterogeneity, the present review is expected to complement previous evidence syntheses and provide a broader assessment of the available randomized evidence.

Beyond providing an updated synthesis of the available evidence, this review is expected to identify priorities for future clinical research, including resveratrol dosage, intervention duration, participant selection, and standardized outcome measures. Depending on the direction, magnitude, consistency, and certainty of the evidence identified [26], the findings may help researchers, clinicians, and guideline developers determine whether the available evidence is sufficient to inform clinical recommendations regarding oral resveratrol supplementation or whether additional randomized controlled trials are required before its routine clinical implementation can be supported. Furthermore, the findings may contribute to future evidence syntheses and help identify areas in which further research is needed to better understand interindividual variability in response to polyphenol supplementation [29].

The present protocol has several methodological strengths, including prospective registration in PROSPERO, adherence to PRISMA-P recommendations, comprehensive searches across multiple databases, independent study selection and data extraction, and standardized assessment of risk of bias and certainty of evidence using the RoB 2 tool and the GRADE approach [19,26,30]. Nevertheless, several anticipated challenges may affect the conduct and interpretation of this review. Expected clinical and methodological heterogeneity among randomized controlled trials—including differences in resveratrol dosage, formulation, duration of supplementation, participant characteristics, and outcome definitions—may limit quantitative synthesis for some outcomes [14,26]. To address these challenges, predefined subgroup analyses, meta-regression (where sufficient data are available), sensitivity analyses, and narrative synthesis will be used as appropriate to explore potential sources of heterogeneity and ensure a transparent interpretation of the evidence. In addition, restricting the review to studies published in English may introduce language bias and affect the comprehensiveness and generalizability of the findings [31]. The restriction to English-language publications was prespecified in the PROSPERO registration and was applied for pragmatic reasons to ensure consistent full-text eligibility assessment and data extraction within the language capabilities and resources available to the review team. Although SciELO indexes the literature in several languages, it was retained as a search source because it also indexes English-language publications from Latin American and other settings that may not be adequately represented in the other databases searched.

Following completion of the review, the findings will be disseminated through publication in a peer-reviewed journal, presentation at scientific conferences, and communication with researchers and health care professionals interested in nutritional interventions for cardiometabolic health. The completed review is also expected to identify evidence gaps and methodological priorities that may guide the design and reporting of future randomized controlled trials evaluating resveratrol supplementation.

In conclusion, this systematic review and meta-analysis is expected to provide an updated and transparent synthesis of the available randomized evidence regarding the effects of oral resveratrol supplementation on inflammatory biomarkers and cardiometabolic outcomes in adults. The findings will help characterize the magnitude, consistency, and certainty of the available evidence, identify relevant sources of clinical and methodological heterogeneity, and highlight remaining evidence gaps. Any implications for clinical practice or future research will be interpreted according to the direction, magnitude, and certainty of the evidence identified in the completed review.

## Supplementary material

10.2196/101301 Multimedia Appendix 1 Complete electronic search strategies, database platforms, and search syntaxes.

10.2196/101301 Checklist 1 PRISMA-P checklist.

## Abbreviations

GRADE: Grading of Recommendations Assessment, Development and Evaluation
IL-6: interleukin-6
PICO: population, intervention, comparator, and outcome
PRISMA: Preferred Reporting Items for Systematic Reviews and Meta-Analyses
PRISMA-P: Preferred Reporting Items for Systematic Review and Meta-Analyses Protocols
RoB 2: risk of bias 2
SWiM: Synthesis Without Meta-analysis
