# Supplementary material for: Effects of Oral Resveratrol Supplementation on Inflammatory and Cardiometabolic Outcomes in Adults: Protocol for a Systematic Review and Meta-Analysis
Source: JMIR Res Protoc. 2026 Oct 27;15:e101301. doi: 10.2196/101301 (PMC13717336; doi:10.2196/101301)
Supplement: Supplementary file 2 [file resprot-v15-e101301-s001.docx]

**Supplementary Material 2**

**Complete Electronic Search Strategies**

**Purpose of the Search Strategy**

This supplementary material provides the complete electronic search strategies developed for the systematic review entitled **"Effects of Oral Resveratrol Supplementation on Inflammatory and Cardiometabolic Outcomes in Adults: Protocol for a Systematic Review and Meta-analysis."**

The search strategy was developed in accordance with the methodological recommendations of the **Preferred Reporting Items for Systematic Reviews and Meta-Analyses Literature Search Extension (PRISMA-S)**, the **Preferred Reporting Items for Systematic Reviews and Meta-Analyses Protocols (PRISMA-P)** statement, and the **Cochrane Handbook for Systematic Reviews of Interventions** to ensure transparency, reproducibility, and methodological rigor.

Following peer-review, the electronic search strategy was comprehensively redesigned to maximize sensitivity and minimize the risk of missing potentially eligible randomized controlled trials. Consistent with current methodological recommendations, the revised strategy combines only two core concepts:

1. **Resveratrol (intervention of interest)**.
2. **Randomized controlled trials**, identified using validated search filters appropriate for each database.

Outcome-related terms, including inflammatory biomarkers, oxidative stress markers, cardiometabolic parameters, anthropometric measures, and other clinical outcomes, were intentionally excluded from the electronic search strategy. Eligibility regarding the predefined primary and secondary outcomes will instead be assessed during title and abstract screening and subsequently confirmed through full-text review according to the predefined eligibility criteria specified in the protocol.

The complete database-specific search strategies presented in this supplementary material constitute the prespecified search methods for the review.

Validated randomized controlled trial filters were used where available (eg, MEDLINE/PubMed), whereas database-specific search strategies recommended by the Cochrane Handbook and PRISMA-S were used for databases and trial registries where validated filters are not available or are not recommended (eg, CENTRAL, Scopus, Web of Science, SciELO, ClinicalTrials.gov, and WHO ICTRP). Minor syntax adjustments required by individual database interfaces may be implemented at the time of search execution without modifying the underlying search concepts, eligibility criteria, or methodological approach. Any such technical adaptations will be transparently documented in the final systematic review.

**Search Concepts**

The electronic searches were constructed using two conceptual blocks only. The first identifies studies evaluating oral resveratrol supplementation, whereas the second identifies randomized controlled trials through validated database-specific filters. The omission of outcome-related terms was intentional to maximize retrieval sensitivity while minimizing the risk of excluding eligible studies due to inconsistent outcome terminology.

**Table S2-1. Search concepts used in the electronic search strategy**

| **Concept** | **Controlled vocabulary** | **Free-text terms** | **Purpose** |
| --- | --- | --- | --- |
| **Resveratrol** | MeSH: *Resveratrol* (MEDLINE); Emtree term where applicable; DeCS term for regional databases | resveratrol; trans-resveratrol | Identification of studies evaluating oral resveratrol supplementation |
| **Randomized controlled trials** | Publication type or database-specific controlled vocabulary for randomized controlled trials, where available | randomized; randomised; randomized controlled trial; controlled clinical trial; placebo; trial; randomly; RCT | Identification of randomized controlled trials using validated search filters |

**Outcome-related terms** (including inflammatory biomarkers, oxidative stress markers, cardiometabolic outcomes, obesity, hypertension, diabetes mellitus, lipid profile, hepatic profile, HOMA-IR, glycated hemoglobin, C-reactive protein, interleukins, tumor necrosis factor-α, total antioxidant capacity, advanced glycation end products, malondialdehyde, DNA damage, and anthropometric variables) were intentionally excluded from the electronic search strategy. These variables constitute predefined eligibility criteria and will be evaluated during study selection rather than during electronic retrieval.

**Information Sources and Search Characteristics**

Electronic searches will be conducted in bibliographic databases and clinical trial registries selected to ensure comprehensive identification of published and ongoing randomized controlled trials evaluating oral resveratrol supplementation in adults.

Search strategies will be adapted to the indexing system and search interface of each information source while preserving the same conceptual structure (Resveratrol + validated randomized controlled trial filter). No restrictions regarding publication year will be applied. Studies published in languages other than English will be identified during the search process but will subsequently be excluded during eligibility assessment according to the predefined inclusion criteria described in the protocol.

**Table S2-2. Information sources and search characteristics**

| **Information source** | **Platform / Provider** | **Source type** | **Controlled vocabulary / indexing** | **Search fields** | **Restrictions applied during search** | **Planned search schedule** |
| --- | --- | --- | --- | --- | --- | --- |
| **MEDLINE** | PubMed (U.S. National Library of Medicine) | Bibliographic database | MeSH terms and free-text keywords | Title/Abstract and MeSH fields | No publication date restrictions; outcome terms excluded | Initial search: August 2026; updated search: January 2027 |
| **CENTRAL** | Cochrane Library (Wiley) | Controlled trials database | Controlled vocabulary where available and free-text keywords | Title, abstract, keywords (ti,ab,kw) | No publication date restrictions; outcome terms excluded | Initial search: August 2026; updated search: January 2027 |
| **Scopus** | Elsevier | Bibliographic and citation database | Free-text keywords | TITLE-ABS-KEY | No publication date restrictions; outcome terms excluded | Initial search: August 2026; updated search: January 2027 |
| **Web of Science Core Collection** | Clarivate Analytics | Bibliographic and citation database | Free-text keywords | Topic (TS) | No publication date restrictions; outcome terms excluded | Initial search: August 2026; updated search: January 2027 |
| **SciELO** | Scientific Electronic Library Online | Regional bibliographic database | DeCS descriptors (where applicable) and free-text keywords | All searchable fields | No publication date restrictions; outcome terms excluded | Initial search: August 2026; updated search: January 2027 |
| **ClinicalTrials.gov** | U.S. National Institutes of Health | Clinical trial registry | Registry indexing and free-text terms | Intervention and keyword fields | Randomized interventional studies | Initial search: August 2026; updated search: January 2027 |
| **WHO International Clinical Trials Registry Platform (ICTRP)** | World Health Organization | International clinical trial registry | Registry indexing and free-text terms | Keyword search | Randomized interventional studies | Initial search: August 2026; updated search: January 2027 |

# The electronic searches will be updated in January 2027, immediately before completion of the review, to identify newly published or registered eligible studies. The final systematic review and meta-analysis is expected to be completed in March 2027. All search dates, database platforms, complete search syntaxes, filters applied, and the number of records retrieved from each information source will be reported in the final publication in accordance with the PRISMA-S reporting guideline.

**Database-Specific Search Strategies**

The electronic search strategies were designed according to the recommendations of the **Cochrane Handbook for Systematic Reviews of Interventions** and the **PRISMA-S Statement**. Search strategies combined controlled vocabulary (when available) with free-text terms to maximize sensitivity. Validated randomized controlled trial filters were used only in databases for which such filters are available and recommended. For pre-filtered databases and clinical trial registries, database-specific strategies were developed without applying additional randomized trial filters.

**MEDLINE (PubMed)**

The MEDLINE search was performed through the PubMed interface using Medical Subject Headings (MeSH), free-text terms, and the **Cochrane Highly Sensitive Search Strategy (HSSS) for identifying randomized controlled trials (PubMed format)**.

```
#1 "Resveratrol"[Mesh]
#2 resveratrol[tiab]
#3 trans-resveratrol[tiab]
#4 #1 OR #2 OR #3
#5 randomized controlled trial[pt]
#6 controlled clinical trial[pt]
#7 randomized[tiab]
#8 placebo[tiab]
#9 drug therapy[sh]
#10 randomly[tiab]
#11 trial[tiab]
#12 groups[tiab]
#13 #5 OR #6 OR #7 OR #8 OR #9 OR #10 OR #11 OR #12
#14 animals[mh] NOT humans[mh]
#15 #13 NOT #14
16. #4 AND #15
```

This strategy corresponds to the **Cochrane Highly Sensitive Search Strategy (sensitivity-maximizing version, PubMed format)**.

**Cochrane CENTRAL**

According to the Cochrane Handbook, **randomized controlled trial filters should not be applied in CENTRAL**, because the database already consists primarily of reports of controlled trials. Consequently, only the intervention concept was searched.

```
#1 MeSH descriptor: [Resveratrol] explode all trees
#2 resveratrol:ti,ab,kw
#3 trans-resveratrol:ti,ab,kw
#4 #1 OR #2 OR #3
```

**Scopus**

Scopus does not have an officially validated Cochrane RCT filter. Therefore, a database-specific strategy based on intervention terms combined with commonly used randomized trial terminology was employed, following Cochrane recommendations for databases lacking validated filters.

TITLE-ABS-KEY (resveratrol OR "trans-resveratrol")

AND

TITLE-ABS-KEY (randomized OR randomised OR placebo OR trial OR randomly OR "controlled clinical trial" OR "randomized controlled trial")

**Web of Science Core Collection**

Searches were conducted using the Topic field (TS), which simultaneously interrogates title, abstract, author keywords and Keywords Plus.

TS=(resveratrol OR "trans-resveratrol")

AND

TS=(randomized OR placebo OR trial OR randomly OR "controlled clinical trial" OR "randomized controlled trial")

**SciELO**

Because SciELO does not provide validated methodological filters for randomized controlled trials, a simplified database-specific strategy was employed.

(resveratrol OR "trans-resveratrol")

AND

(randomized OR randomised OR placebo OR trial OR ensaio OR ensayo)

**ClinicalTrials.gov**

ClinicalTrials.gov is an international registry of clinical studies; therefore, no methodological filter for randomized controlled trials was applied.

Condition/Disease: Any

Other Terms: resveratrol OR trans-resveratrol

Study Type: Interventional Studies

Allocation: Randomized

Only randomized interventional studies evaluating oral resveratrol supplementation in adults will be considered during eligibility assessment.

**WHO International Clinical Trials Registry Platform (ICTRP)**

The ICTRP is an international registry that aggregates randomized and non-randomized interventional studies from multiple primary registries. Consequently, no additional randomized trial filter was applied.

Resveratrol OR trans-resveratrol

Study design eligibility (randomized controlled trial) will be determined during title screening and registry record assessment according to the predefined eligibility criteria.

**Additional Search Procedures**

To maximize the completeness of evidence identification and minimize the risk of publication bias, additional search procedures will be undertaken following completion of the electronic database searches.

**Backward Citation Searching**

Reference lists of all studies included after full-text screening, as well as relevant systematic reviews identified during the selection process, will be manually examined to identify additional eligible randomized controlled trials not retrieved through the electronic database searches.

**Forward Citation Tracking**

Forward citation searching will be conducted using citation indexes available through **Scopus** and **Web of Science Core Collection** to identify more recent studies citing eligible randomized controlled trials.

**Clinical Trial Registries**

Clinical trial registries will be searched independently to identify completed but unpublished studies, ongoing randomized controlled trials, and studies with results pending publication.

The following registries will be searched:

- ClinicalTrials.gov
- World Health Organization International Clinical Trials Registry Platform (WHO ICTRP)

When multiple records correspond to the same clinical trial, registry entries will be linked to the corresponding published reports whenever possible.

**Contact with Study Authors**

When essential methodological information or outcome data required for eligibility assessment or quantitative synthesis are unavailable or unclear, the corresponding authors of eligible studies will be contacted by email.

Up to two contact attempts will be made over a four-week period.

If no response is received, the study will not be excluded solely because additional information could not be obtained. Available published data will be used, and any remaining uncertainty will be considered during the risk of bias assessment and sensitivity analyses.

**Duplicate Record Management**

All retrieved references will be exported into a reference management software package for automatic duplicate detection.

Subsequently, duplicate records will be manually reviewed to ensure accurate identification of unique studies before title and abstract screening.

**Search Update**

To ensure that the systematic review incorporates the most recent available evidence, all electronic searches will be updated immediately before completion of the review.

The updated searches will use the identical search strategies presented in this supplementary material.

Only studies published or registered after completion of the initial search will be screened during the update process.

The planned schedule is as follows:

| **Activity** | **Planned date** |
| --- | --- |
| Initial electronic searches | August 2026 |
| Updated electronic searches | January 2027 |
| Completion of study selection | February 2027 |
| Completion of systematic review and meta-analysis | March 2027 |

If newly identified studies satisfy the predefined eligibility criteria, they will undergo the same screening, data extraction, risk of bias assessment, and statistical analysis procedures as studies identified during the initial search.

**Protocol Amendments Related to the Search Strategy**

Following peer review, the electronic search strategy was revised to align with current recommendations from the Cochrane Handbook and PRISMA-S. The revised strategy removed outcome-related search terms and incorporated validated randomized controlled trial filters where appropriate. These revisions did not modify the review question, eligibility criteria, or planned analytical methods.

**References**

The following methodological references guided the development of the electronic search strategy and reporting procedures.

1. Higgins JPT, Thomas J, Chandler J, Cumpston M, Li T, Page MJ, Welch VA, editors. **Cochrane Handbook for Systematic Reviews of Interventions. Version 6.5.1**. Cochrane; 2025.
2. Rethlefsen ML, Kirtley S, Waffenschmidt S, Ayala AP, Moher D, Page MJ, et al. **PRISMA-S: an extension to the PRISMA Statement for Reporting Literature Searches in Systematic Reviews.** *Systematic Reviews*. 2021;10:39.
3. Page MJ, Moher D, Bossuyt PM, Boutron I, Hoffmann TC, Mulrow CD, et al. **PRISMA 2020 statement: an updated guideline for reporting systematic reviews.** *BMJ*. 2021;372:n71.
4. Moher D, Shamseer L, Clarke M, Ghersi D, Liberati A, Petticrew M, et al. **Preferred Reporting Items for Systematic Review and Meta-Analysis Protocols (PRISMA-P) 2015 statement.** *Systematic Reviews*. 2015;4:1.
5. Lefebvre C, Glanville J, Briscoe S, Littlewood A, Marshall C, Metzendorf MI, Noel-Storr A, Rader T, Shokraneh F, Thomas J, Wieland LS. **Chapter 4: Searching for and selecting studies.** In: *Cochrane Handbook for Systematic Reviews of Interventions*. Version 6.5.1. Cochrane; 2025.
